# Microneedle‐Delivered PDA@Exo for Multifaceted Osteoarthritis Treatment via PI3K‐Akt‐mTOR Pathway

**DOI:** 10.1002/advs.202406942

**Published:** 2024-08-29

**Authors:** Zihua Li, Hengli Lu, Limin Fan, Xiaoyi Ma, Zhengwei Duan, Yiwei Zhang, Yuesong Fu, Sen Wang, Yonghao Guan, Dong Yang, Qingjing Chen, Tianyang Xu, Yunfeng Yang

**Affiliations:** ^1^ Department of Orthopaedics Ruijin Hospital Shanghai Jiao Tong University School of Medicine Shanghai 200025 P. R. China; ^2^ Department of Orthopedics Shanghai Tenth People's Hospital School of Medicine Tongji University Shanghai 200072 P. R. China; ^3^ School of Medicine Tongji University Shanghai 200092 P. R. China; ^4^ Southern Medical University Guangzhou 510515 P. R. China

**Keywords:** macrophage polarization, microneedle drug delivery, osteoarthritis, polydopamine‐exosome complex, reactive oxygen species scavenging

## Abstract

Osteoarthritis (OA) is marked by cartilage deterioration, subchondral bone changes, and an inflammatory microenvironment. The study introduces the Microneedle‐Delivered Polydopamine‐Exosome (PDA@Exo MN), a therapeutic that not only preserves cartilage and promotes bone regeneration but also improves localized drug delivery through enhanced penetration capabilities. PDA@Exo MN shows strong reactive oxygen species (ROS) scavenging abilities and high biocompatibility, fostering osteogenesis and balancing anabolic and catabolic processes in cartilage. It directs macrophage polarization from M0 to the anti‐inflammatory M2 phenotype. RNA sequencing of treated chondrocytes demonstrates restored cellular function and activated antioxidant responses, with modulated inflammatory pathways. The PI3K‐AKT‐mTOR pathway's activation, essential for PDA@Exo's effects, is confirmed via bioinformatics and Western blot. In vivo assessments robustly validate that PDA@Exo MN prevents cartilage degradation and OA progression, supported by histological assessments and micro‐CT analysis, highlighting its disease‐modifying impact. The excellent biocompatibility of PDA@Exo MN, verified through histological (H&E) and blood tests showing no organ damage, underscores its safety and efficacy for OA therapy, making it a novel and multifunctional nanomedical approach in orthopedics, characterized by organ‐friendliness and biosecurity.

## Graphical abstract

Introducing Microneedle‐Delivered Polydopamine‐Exosome (PDA@Exo MN), a novel osteoarthritis therapeutic with enhanced drug delivery via improved penetration. Demonstrating strong antioxidative properties and high biocompatibility, PDA@Exo MN preserves cartilage, promotes bone regeneration, and effectively modulates inflammatory pathways to halt disease progression.

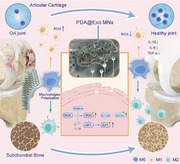

## 1 Introduction

OA is a prevalent and debilitating condition that is becoming more common due to aging, rising obesity rates, and an increase in joint injuries. Worldwide, it is estimated that 250 million people currently suffer from osteoarthritis, posing significant health and socioeconomic burdens.^[^ ^1^ ^]^ Currently, there are no non‐surgical interventions that can prevent, halt, or slow down the progression of osteoarthritis.^[^ ^2^ ^]^ Pharmaceutical therapies, such as nonsteroidal anti‐inflammatory drugs (NSAIDs), primarily focus on pain relief and anti‐inflammation. However, they only offer limited control over osteoarthritis symptoms and cannot reverse joint damage. Furthermore, the effectiveness of these drugs can be compromised by the harsh gastrointestinal environment and the liver's first‐pass effect, which can result in significantly low drug bioavailability.^[^ ^3^ ^]^ Therefore, it is important to develop new treatments and effective anti‐inflammatory drugs for OA to reduce social burden associated with OA.^[^ ^4^ ^]^

OA is a chronic disease caused by joint inflammation, resulting from damage to the articular cartilage and degradation of extracellular matrix components such as collagen II and proteoglycan.^[^ ^5^ ^]^ Activated macrophages are a major contributor to inflammatory responses in OA joints, producing significant amounts of ROS which can induce extracellular toxicity and degradation of cartilage matrix.^[^ ^6^ ^]^ Prolonged ROS presence can amplify pro‐inflammatory pathways, activate macrophages, and worsen inflammation. Targeting the proliferation of activated macrophages and reducing existing ROS levels can reduce inflammation.^[^ ^7^ ^]^ Additionally, ROS can be used to induce oxidation‐responsive release of anti‐inflammatory drugs, further enhancing therapeutic effects in OA.^[^ ^8^ ^]^ Efforts to decrease levels of ROS have focused on creating an effective treatment approach that applies multifunctional biomaterials, which can both reduce oxidative stress and promote chondrogenesis. Besides, OA is a complex disease involving multiple pathways, including inflammation via macrophages, although the precise role and mechanisms of macrophages in OA remain unknown. Macrophages differentiate into M1 and M2 categories in response to microenvironment stimuli, with M1 macrophages secreting proinflammatory cytokines and M2 macrophages having anti‐inflammatory activity.^[^ ^8^, ^9^ ^]^ The accumulation and polarization of macrophages in the synovium and articular cavity during OA development suggest a correlation, but recent studies show that macrophage depletion, reducing both M1 and M2, increases inflammation and fails to alleviate OA severity.^[^ ^10^ ^]^ However, current biomaterial treatments for OA mainly encapsulate growth factors or deliver antioxidants, without comprehensively addressing the complex process of preventing OA. Therefore, the development of new biomaterial tools with multiple functions for OA treatment is still highly anticipated.

Microneedle patches have functional designs that make them highly effective in delivering drugs to specific sites in various disease microenvironments, such as solid tumors and organs. Their ability to penetrate a variety of tissues and organs makes them an ideal biomaterial for lesion positioning therapy in medicine. In this paper, we present a novel core‐shell MN patch with mesenchymal stem cell (MSC)‐derived exosomes (Exo) and polydopamine nanoparticles (PDA NPs) encapsulated in the needle tips for OA, as schemed 1. Transdermal drug delivery has the advantage of avoiding gastrointestinal digestion associated with oral routes and pain sensations caused by injections and the ease of self‐administration increases patients’ compliance.^[^ ^11^ ^]^ Furthermore, the designed MN patch could be integrally degradable and biocompatible, which made the drug delivery and absorption more efficient, convenient, and safer.^[^ ^12^ ^]^ While traditional MNs are limited in function, MSC‐derived exosomes offer a promising approach for treatment by leveraging their nanoscale size and advanced functions inherited from parent stem cells.^[^ ^13^ ^]^ Besides, MSC Exos, created by extruding MSCs through porous membranes, offer a 250‐fold increase in yield production and enhanced expression of mRNAs and proteins, making them highly valuable for practical clinical applications. Despite their successes, the expression amount and level of therapeutic growth factors in MSC NVs are still limited, requiring further development to meet the demands of more effective efficacy.^[^ ^14^ ^]^

To counteract the negative effects of ROS‐derived oxidative stress, PDA NPs, which contain reductive functional groups like catechol and imine, were encapsulated in the outer methacrylated hyaluronic acid (HAMA) shell of MN tips, as PDA has been proven to be an effective antioxidant agent.^[^ ^15^ ^]^ This sustained release of PDA NPs from degrading HAMA patch tips effectively suppresses the ROS‐induced inflammation reaction at the lesion site. In addition, Exo MN have been shown to significantly increase cell proliferation as well as inhibit cell apoptosis, while the combination of PDA NPs and Exos can further promote M2 macrophage polarization, effectively suppressing inflammation. Collectively, the Exo PDA@MN patch has demonstrated excellent anti‐inflammatory, antioxidant, and promote osteogenesis, making it highly effective for delaying the progression of OA, which highlights the potential of the proposed composite core‐shell MN patch as a promising candidate for OA treatment.

## 2 Results and Discussion

### 2.1 Preparation and Characterization of MSC‐Exos and PDA NPs

**Figure 1:**
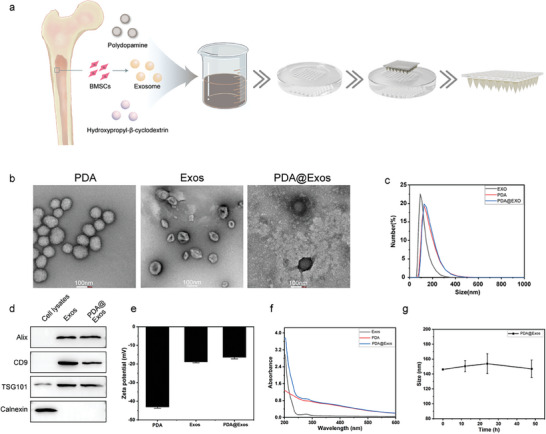
Synthesis and characterization of PDA@Exo MN. a) Diagrammatic representation of the synthesis pathway for PDA@Exo MN. b) Transmission electron microscopy (TEM) images depicting PDA, Exo, and PDA@Exo. Scale bars: 100 µm. c) Hydrodynamic diameters of Exo, PDA and PDA@Exo determined by Dynamic Light Scattering (DLS). d) Western blot analysis of exosome markers (Alix, CD9, TSG101) and endoplasmic reticulum (ER) markers (calnexin). e) Zeta potential measurements of PDA, Exo, and PDA@Exo. f) UV spectra of PDA, Exo, and PDA@Exo. g) Evaluation of the size stability of PDA@Exo.

The synthetic route of PDA NPs is shown in **Figure** **1**a. In a typical experiment, MSCs were co‐cultured with NPs for 24 h, and the MSCs were isolated by ultracentrifugation following established protocol (Figure S1, Supporting Information).^[^ ^16^ ^]^ TEM images reveal that PDA nanoparticles exhibit a spherical structure with a diameter of ≈100 nm, while Exo microstructure appears as smooth spheres, and the morphology of PDA@Exo composite material is consistent with that of PDA nanoparticles, also exhibiting a spherical structure (Figure 1b). Western blot analysis was utilized to detect exosome markers (Alix, CD9, TSG101) and endoplasmic reticulum (ER) markers (calnexin) present in PDA@Exo exosomes released by cells (Figure 1c). Additionally, DLS analysis reveals that PDA nanoparticles and PDA@Exo composite material both have a narrow size distribution with diameters of ≈108 and 106 nm respectively, consistent with TEM observations (Figure 1d) and further supported by Nanoparticle Tracking Analysis (NTA) of the exosomes and PDA@Exo composites (Figure S2, Supporting Information). Zeta potential and ultraviolet tests were conducted to confirm the successful synthesis of PDA@Exo composite material, revealing significant changes in its potential value (Figure 1e) compared to individual PDA and Exo nanoparticles. The UV spectrum of the PDA@Exo composite material (Figure 1f) also exhibited characteristic peaks of Exo extracellular vesicles, distinguishing it from the PDA group. Furthermore, the stability of PDA@Exo nanoparticles for 48 h (Figure 1g) suggests their potential applicability for sustained drug delivery and therapeutic applications.

### 2.2 Preparation and Characterization of Core–Shell MN Patch

**Figure 2:**
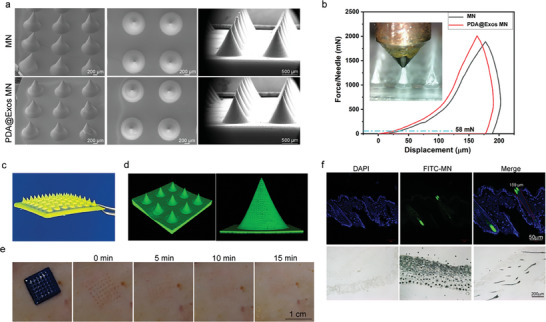
Microstructural Analysis and Mechanical Validation of Microneedles. a) SEM Micrographs of MN and PDA@Exo MN. b) Mechanical Characterization of Microneedles: MN versus PDA@Exo MN. c) Photomicrograph of a fluorescent MN. d) 3D reconstruction image of Laser Confocal Microscopy Image of a Microneedle. e) Sequential evaluation of skin recovery at various time points post‐microneedle insertion. Scale bars: 1 cm. f) Immunofluorescent and histological staining of MN penetration in rat skin. Scale bars: 50 and 200 µm, respectively.

The successful coating with the PDA@Exo composite, evident from the increased surface roughness in **Figure** **2**a, is highlighted through SEM imaging, which also shows the maintained uniform conical geometry of the microneedle arrays. Notably, the microneedles manifest a distinctive conical shape, characterized by uniform dimensions and remarkably sharp tips, which collectively meet the essential geometric criteria required for optimal and efficient skin penetration. These microneedles exhibit a remarkable height of 1000 ± 10 micrometers, a base width of 900 ± 10 micrometers and a diameter of 904.06 µm in blank MN and 879.36 µm, respectively (Figures S3 and S4, Supporting Information). The analysis of the force‐displacement curve demonstrates that the fracture force of the microneedles significantly exceeds the skin's minimum penetration threshold of 58 mN, confirming their effectiveness for reliable skin penetration. Moreover, the incorporation of the PDA@Exo composite material leads to a noticeable increase in the microneedles' mechanical strength, as evidenced by the enhanced slope in the force‐displacement curve within the 20–40% range, indicative of improved mechanical properties (Figure 2b). Additionally, Figure 2c displays a micrograph of the microneedle array with 49 needles organized in a precise 7 × 7 arrangement, highlighted by uniform FITC dye fluorescence, signifying even coating distribution. A 3D reconstructed image from laser confocal microscopy offers a detailed perspective of the microneedles’ topography and precise architecture, as shown in Figure 2d. Upon application to rat skin, water‐soluble microneedles demonstrate a swift dissolution and delivery of compounds, with the skin exhibiting rapid healing within minutes, as the puncture marks nearly vanish by the 15 min observation, underscoring a quick recovery and the non‐invasive nature of the delivery system (Figure 2e). Histological analyses confirm the patches’ ability to penetrate various epidermal layers, reaching up to 159 µm into the dermis and thereby reducing discomfort by avoiding deeper, more sensitive tissue areas (Figure 2f). To further assess transdermal penetration, PKH26‐labeled EXOs/MN were applied to fresh porcine skin; fluorescence imaging of frozen skin sections demonstrated that EXOs could penetrate deeply, up to 800 µm into the skin (Figure S5, Supporting Information). The in vitro release of EXOs from microneedles was evaluated using a BCA protein assay in a 24‐well Transwell setup, where the cumulative release rate reached 58.85% by 12 h and then stabilized, indicating effective and controlled delivery (Figure S6, Supporting Information). Additionally, methylene blue‐labeled microneedles release their payload rapidly upon insertion into agar gel and achieve a stable release over 7 days, suggesting their suitability for sustained drug delivery and thus, this could enhance compliance by reducing the frequency of dosing, making them promising for chronic condition management (Figure S7, Supporting Information).

### 2.3 Antioxidant Effect of PDA@Exo Nanoparticles against ROS

**Figure 3:**
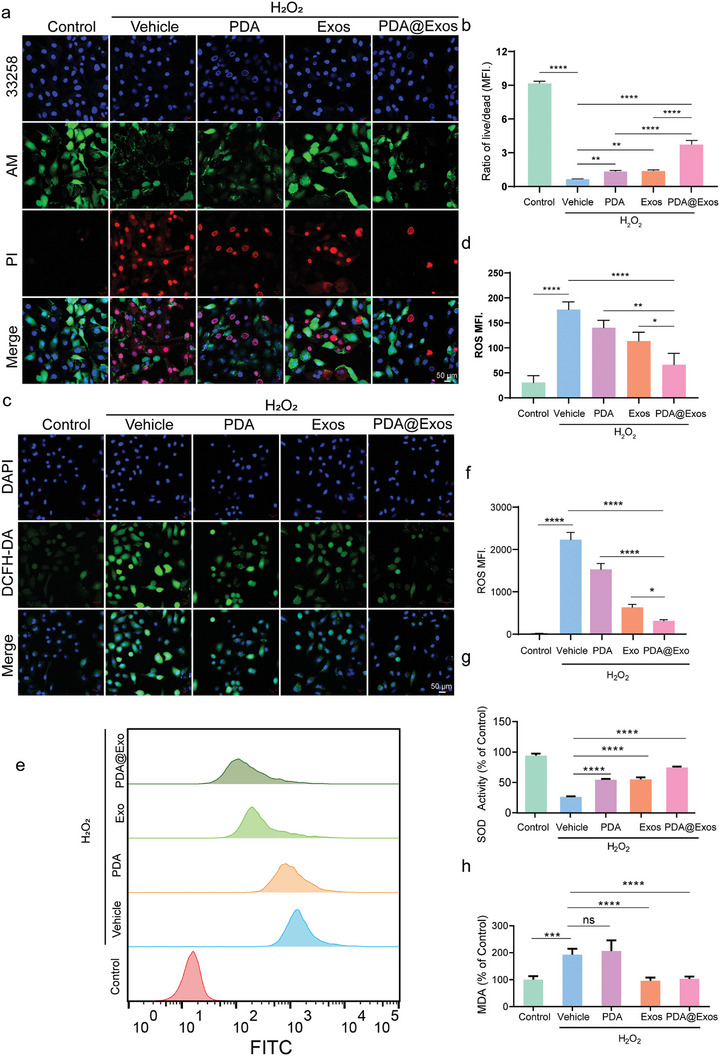
Protective effect of PDA@Exo MN on chondrocytes against ROS in vitro. a) Live/Dead staining of chondrocytes treated with Control, PDA, Exo, and PDA@Exo under H_2_O_2_ stimulation, showing nuclei in blue (33258), live cells in green (AM) and dead cells in red (PI). Scale bars: 20 µm. b) Ratio of live/dead cells measured by MFI in chondrocytes post‐treatment with Control, PDA, Exo, and PDA@Exo under H_2_O_2_ stimulation. c) ROS detection in chondrocytes treated with Control, PDA, Exo, and PDA@Exo under H_2_O_2_ stimulation, where green fluorescence (DCF) indicates ROS levels, with DAPI staining for nuclei. Scale bars: 50 µm. d) Quantitative analysis of intracellular ROS levels as indicated by mean fluorescence intensity (MFI) in chondrocytes treated with Control, PDA, Exo, and PDA@Exo following H_2_O_2_ stimulation. e) ROS detection using FITC in chondrocytes treated with Control, PDA, Exo, and PDA@Exo under H_2_O_2_ stimulation. f) MFI in chondrocytes treated with Control, PDA, Exo, and PDA@Exo following H_2_O_2_ stimulation. g) Relative SOD activity restored by PDA@Exo in H_2_O_2_‐induced chondrocytes. h) Cellular MDA content of chondrocytes treated with Control, PDA, Exo, and PDA@Exo under H_2_O_2_ stimulation All data are shown as the mean ± standard deviation (SD). *p < 0.05, **p < 0.01, ***p < 0.001.

To ensure safe use for bio‐applications, the cytotoxicity of PDA@Exo was evaluated using a cell counting kit‐8 (CCK‐8) assay on primary rat chondrocytes. Results showed no significant decrease in cell viability after 48 h of incubation with PDA@Exo at concentrations up to 400 µg mL^−1^, indicating minimal cytotoxicity (Figure S8, Supporting Information). Subsequently, chondrocytes were pre‐treated with H_2_O_2_ (800 µm) for 24 h to confirm the protective effect of PDA@Exo against cellular ROS damage by Live/dead cell double staining using calcein‐acetoxymethyl ester/propidium iodide (Calcein‐AM/PI) kit. The results showed that PDA@Exo efficiently protected chondrocytes from H_2_O_2_‐induced cell death, as evidenced by the significant increase in live cells after PDA@Exo addition compared to the almost complete cell death in the control group (**Figure** **3**a,b). Studies have demonstrated excessive production of ROS and oxidative stress in chondrocytes are major contributors to the pathogenesis of OA.^[^ ^17^ ^]^ Under pathological conditions in OA, excessive ROS act as secondary messengers, promoting cartilage degradation by inducing matrix‐degrading proteases, reducing ECM synthesis, and inducing chondrocyte apoptosis.^[^ ^18^ ^]^ Therefore, leveraging PDA NPs, renowned for their ROS scavenging capabilities in conditions such as tumors, infections, and inflammatory diseases including OA, may offer therapeutic potential by diminishing ROS production and impeding cartilage matrix catabolism.^[^ ^19^ ^]^ Additionally, numerous studies have demonstrated that MSCs‐derived exosomes possess the ability to repair damaged tissues, protect cells, regulate immune responses, and inhibit inflammation.^[^ ^20^ ^]^ Intracellular ROS levels were quantitatively assessed using the ROS‐sensitive fluorescent probe DCFH‐DA, which diffuses into cells and fluoresces upon oxidation, with flow cytometry providing quantitative measurement of oxidation levels. Enhanced green fluorescence indicative of increased ROS was observed in chondrocytes subjected to oxidative stress by H_2_O_2_. Notably, the application of PDA@Exos markedly attenuated the H_2_O_2_‐induced ROS levels, as evidenced by reduced fluorescence intensity in the treated samples (Figure 3c,d). FITC results corroborated the observed decrease in ROS, quantitatively confirming a significant reduction in median fluorescence intensity, which illustrates the effective suppression of ROS by PDA@Exos in treated cells (Figure 3e,f). Superoxide dismutase (SOD) serves as a crucial defensive enzyme against oxidative stress by neutralizing superoxide anions, while malondialdehyde (MDA) levels indicate the extent of lipid peroxidation by ROS. In this study, PDA@Exos was found to significantly restore SOD activity diminished by H_2_O_2_ exposure in chondrocytes, suggesting a recovery of the cells' antioxidative mechanisms. Additionally, PDA@Exos effectively reduced the elevated MDA content, indicating a decrease in lipid peroxidation and an overall reduction of intracellular ROS levels (Figure 3g,h). Enzymes like SOD3, GPX, and CAT play essential roles in cellular defenses against oxidative stress by regulating the balance of ROS. PDA@Exos treatment was observed to significantly enhance the expression of these enzymes, suggesting its supportive role in reinforcing the cell's antioxidative capacity. Concurrently, PDA@Exos also reduced the mRNA expression of iNOS and COX‐2, inflammatory markers linked to cartilage damage, highlighting its dual protective effect against both oxidative stress and inflammation in chondrocytes (Figure S9, Supporting Information). These results collectively highlight PDA@Exo as a potent ROS scavenger, capable of preventing cartilage deterioration and thereby emerging as a promising candidate for OA therapy.

### 2.4 Therapeutic Modulation of Chondrocyte Function and Macrophage Polarization by PDA@Exo In Vitro

**Figure 4:**
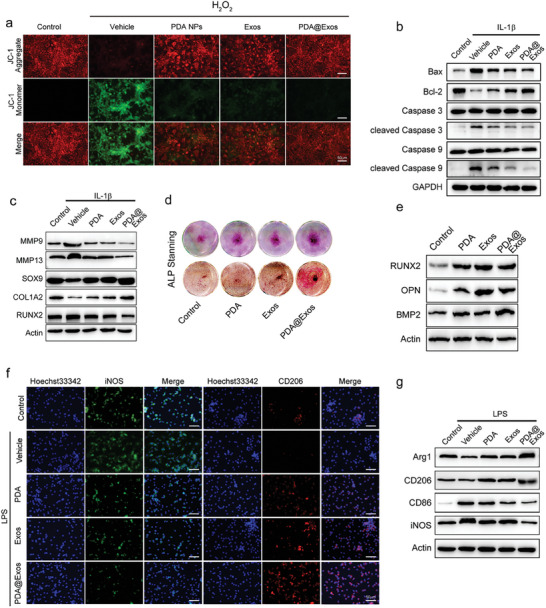
PDA@Exo restore the cellular function of chondrocytes under oxidative stress and M2 polarization. a) JC‐1 staining of chondrocytes treated with Control, PDA, Exo, and PDA@Exo under H_2_O_2_ stimulation to monitor mitochondrial membrane potential. Scale bars: 50 µm. b) Western blot analysis of the expression of apoptosis markers in chondrocytes treated with Control, PDA, Exo, and PDA@Exo under IL‐1β stimulation. c) Western blot analysis of the expression of MMP9, MMP13, SOX9, COL1A2, and RUNX2 in chondrocytes treated with Control, PDA, Exo, and PDA@Exo under IL‐1β stimulation. d) Alizarin red S staining of BMSCs treated with Control, PDA, Exo, and PDA@Exo. e) Western blot analysis of the expression of osteogenesis‐related genes (RUNX2, OPN, and BMP2) in BMSCs treated with Control, PDA, Exo, and PDA@Exo. f) Immunofluorescence staining of the RAW 264.7 cells after being cocultured with control, PDA, Exos and PDA@Exo under LPS stimulation, iNOS (green); CD206 (red); Blue: nuclei. Scale bars: 50 µm. g) Western Blot analysis of RAW 264.7 cells after being cocultured with control, PDA, Exos and PDA@Exo under LPS stimulation. (Scale bars: 50 µm) All data are shown as the mean ± standard deviation (SD). *p < 0.05, **p < 0.01, ***p < 0.001.

Given that mitochondria constitute a principal source of intracellular ROS and serve as the epicenter of energy metabolism, they inherently become susceptible targets for ROS‐induced cellular impairment. To evaluate the influence of PDA@Exo on the mitochondrial function of chondrocytes, we employed a JC‐1 mitochondrial membrane potential assay, wherein well‐functioning mitochondria with high membrane potential emit red fluorescence due to JC‐1 aggregation, while dysfunctional mitochondria with low membrane potential emit green fluorescence due to JC‐1 monomers. Healthy chondrocytes exhibited pronounced red fluorescence, which was significantly diminished upon exposure to H_2_O_2_, leading to an increase in green fluorescence; the presence of PDA@Exo significantly elevated the aggregate to A/M fluorescence ratio, which had been sharply reduced by H_2_O_2_ exposure, indicating a substantial mitigation of mitochondrial dysfunction compared to groups treated with PDA or Exo alone (**Figure** **4**a; Figure S10, Supporting Information). Furthermore, the ATP content within chondrocytes, a direct measure of mitochondrial function, was notably compromised by H_2_O_2_, with PDA@Exo leaching solution effectively restoring ATP production, indicating the potential of PDA@Exo to support cellular energy requirements under oxidative stress (Figure S11, Supporting Information). Furthermore, to investigate the protective effect of PDA@Exo on chondrocyte apoptosis, the expression levels of Bax, Bcl‐2, Caspase 3, cleaved Caspase 3, Caspase 9 and cleaved Caspase 9, were examined using western blot analysis.^[^ ^21^ ^]^ The results showed that IL‐1β treatment significantly upregulated the expression of Bax, cleaved Caspase 3 and cleaved caspase 9 and while downregulating the expression of Bcl‐2, suggesting an induction of chondrocyte apoptosis.^[^ ^22^ ^]^ However, pretreatment with PDA@Exo resulted in a significant decrease in the expression of Bax, cleaved caspase 3 and cleaved caspase 9 while increasing the expression of Bcl‐2 comparing to control group, indicating a suppression of chondrocyte apoptosis (Figure 4b; Figure S12, Supporting Information). RT‐PCR analysis of critical cartilage matrix anabolic genes—including type II collagen (COL2A1), aggrecan (ACAN), and SRY‐box 9 (SOX9)—revealed that their suppressed expression in IL‐1β‐treated chondrocytes was significantly reversed by treatments with PDA@Exo and PDA@Exo, indicating a protective role against chondrocyte dysfunction in OA. This therapeutic potential was further highlighted by the inhibition of the upregulation of Adamts5, Adamts1, and MMP13, genes pivotal to cartilage matrix degradation, suggesting an inhibitory effect of PDA@Exo on catabolic processes within OA chondrocytes (Figure S13, Supporting Information). To investigate the protective effect of PDA@Exo on chondrocytes in an IL‐1β‐induced model, the expression levels of MMP9, MMP13, SOX9, COL1A2 and RUNX2 were examined using western blot analysis (Figure 4c; Figure S14, Supporting Information).^[^ ^23^ ^]^ IL‐1β induced model remarkably increased the levels of MMP9 and MMP13 while chondrocytes co‐treated with PDA@Exo resulted in decreased levels of MMP9 and MMP13, which are known to be involved in cartilage degradation. Furthermore, RUNX2 expression was downregulated by PDA@Exo, indicating a protective effect against hypertrophy.^[^ ^24^ ^]^ PDA@Exo increased the expression levels of SOX9 and COL1A2, which are important markers of chondrogenic differentiation.^[^ ^25^ ^]^ In addition, the osteogenic potential of BMSCs, assessed via Alizarin red staining after 21 days of culture in osteogenic induction medium with or without PDA@Exo, demonstrated an enhanced formation of calcium nodules in the PDA@Exo groups (Figure 4d). This was indicative of a bolstered osteogenic capability, further corroborated by elevated protein levels of osteogenesis‐associated genes (OPN, RUNX2, and BMP2), underscoring the contribution of PDA@Exo to subchondral bone repair in vitro (Figure 4e; Figure S15, Supporting Information). Overall, PDA@Exo can protect chondrocytes by inhibiting apoptosis and promoting extracellular matrix synthesis, suggesting its potential as a therapeutic strategy for the management of osteoarthritis.

Macrophage polarization is a crucial factor in the development and progression of osteoarthritis. An imbalance toward M1 macrophages leads to inflammation and cartilage degradation, while M2 macrophages promote cartilage repair through the secretion of anti‐inflammatory cytokines and growth factors, making M2 polarization a promising therapeutic strategy for osteoarthritis.^[^ ^26^ ^]^ To investigate the potential antioxidative and anti‐inflammatory effects of PDA@Exo on macrophage polarization in osteoarthritis, we incubated PDA, Exo and PDA@Exo with 50 ng mL^−1^ lipopolysaccharide (LPS)‐stimulated RAW264.7 cells. Additionally, immunofluorescence staining of Vihecle, PDA, Exo and PDA@Exo with LPS‐stimulated RAW264.7 cells revealed that iNOS expression was significantly decreased in PDA@Exo groups and CD206 was increased compared to the levels in the control group (Figure 4f; Figure S16, Supporting Information). Post‐incubation with PDA@Exo, western blot analysis of RAW264.7 cells revealed a notable upregulation in M2 macrophage markers, Arg1 and CD206, and a concurrent downregulation of the M1 marker iNOS, indicating a shift toward anti‐inflammatory phenotype (Figure 4g; Figure S17, Supporting Information). ELISA results revealed that PDA@Exos effectively attenuates LPS‐induced increases in pro‐inflammatory cytokines IL‐6, TNF‐α, and IL‐1β, highlighting its potential to counteract M1 macrophage‐mediated inflammation (Figure S18, Supporting Information). These findings suggest that PDA@Exo treatment can induce M2 polarization of macrophages, consistent with the observed anti‐inflammatory effects. Since the M1/M2 macrophage balance plays a critical role in the progression of various diseases, including osteoarthritis, our study demonstrated that PDA@Exo treatment can effectively shift the balance toward the M2 phenotype, indicating its potential as a therapeutic strategy for osteoarthritis.

### 2.5 Transcriptomic Analysis of PDA@Exo against IL‐1β Induced OA Chondrocytes

**Figure 5:**
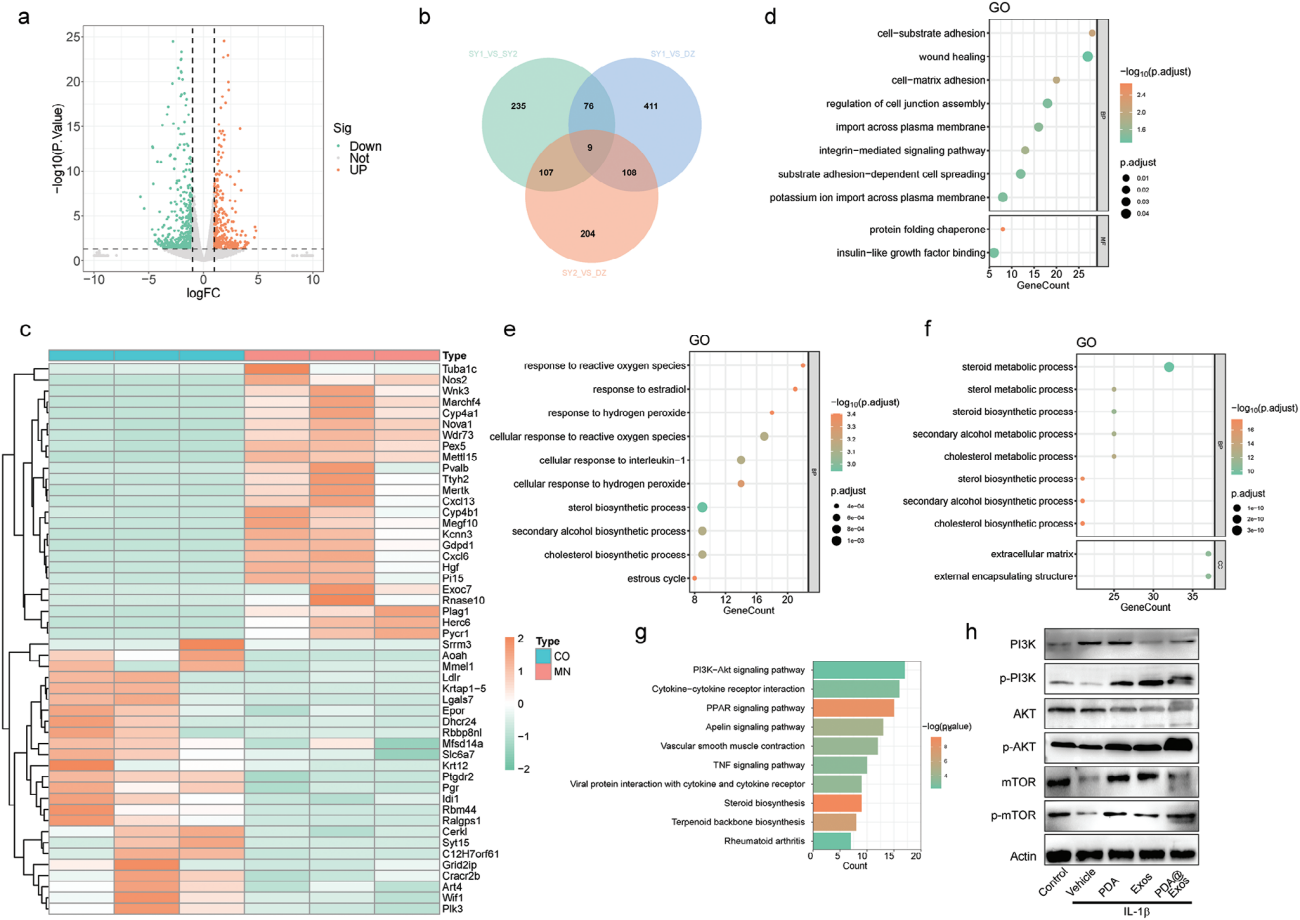
Changes in transcriptome profile of PDA@Exo ‐treated OA chondrocytes.a) The volcano plot of differentially expressed genes (DEGs) and b) Venn that are essential for chondrocyte bio‐function and provide cellular protection against inflammation induced by IL‐1β are identified and highlighted. c) Heatmap representing the gene expression profiles of OA chondrocytes stimulated with IL‐1β and OA chondrocytes treated with IL‐1β + PDA@Exo. d–f) The results of Gene Ontology (GO) enrichment analysis in both up‐regulated and down‐regulated genes. g) Enrichment analysis of the KEGG pathways for the differentially expressed genes of interest. h) The expression of p‐PI3K, PI3K, p‐Akt, Akt, p‐mTOR, and mTOR protein in chondrocyte treated with control, PDA, Exos and PDA@Exo under IL‐1β stimulation.

Building on our previous promising results, we extended our research to perform RNA sequencing analysis, aiming to understand the effects of PDA@Exo on chondrocytes in osteoarthritis (OA). After RNA sequencing, **Figure** **5**a shows a volcano plot identifying differentially expressed genes, with significant upregulation and downregulation noted byFDR < 0.05, log2FC > 1, or log2FC < −1. A Venn diagram in Figure 5b illustrates the overlap of gene expression changes between conditions, highlighting a core set of affected genes among control, OA group and OA with PDA@Exo MN treatment group. Additionally, the heatmap details gene expression levels across samples, with hierarchical clustering indicating distinct expression profiles, as decoded by the legend (Figure 5c). To delve deeper into the affected biological functions and pathways, we carried out Gene Ontology (GO) enrichment analysis and Kyoto Encyclopedia of Genes and Genomes (KEGG) pathway enrichment analysis. Our GO analysis highlights PDA@Exo's anti‐inflammatory role in IL‐1β‐stimulated chondrocytes, showing an upregulation of antioxidative responses, modulation of lipid biosynthesis, and enhancement of extracellular matrix integrity. Notably, the upregulation of “response to hydrogen peroxide” and “response to reactive oxygen species” underscores an enhanced antioxidative defense mechanism. Simultaneously, the activation of “cholesterol biosynthetic process” and “sterol biosynthetic process” signals a reparative shift in lipid metabolism. Additionally, the enrichment of terms like “extracellular matrix” and “collagen‐containing extracellular matrix” indicates an improvement in tissue repair and resilience to inflammation. These findings highlight PDA@Exo's role in dampening IL‐1β‐mediated inflammatory pathways, showcasing its potential for osteoarthritis therapy (Figure 5d,e). The KEGG pathway enrichment analysis further revealed a significant modulation of the PI3K‐Akt signaling pathway in chondrocytes treated with PDA@Exo post IL‐1β stimulation (Figure 5g). This pathway, crucial for various cellular functions such as growth, survival, and inflammation, showed significant gene expression changes, suggesting PDA@Exo's ability to mitigate osteoarthritis pathophysiology by modulating the PI3K‐Akt pathway. Moreover, related inflammatory pathways like TNF and IL‐17 signaling were also modulated, with a notable emphasis on the PI3K‐Akt pathway's role.The downstream effects of this modulation hint at a potential reduction in pro‐inflammatory mediators alongside an improvement in cellular homeostasis. These insights reveal the complex impact of PDA@Exo, especially highlighting the significant role of the PI3K‐Akt pathway in managing chondrocyte pathophysiology amidst inflammatory stress. The extensive changes in gene expression related to this pathway highlight its therapeutic promise for mitigating inflammation in osteoarthritis.

To validate the anti‐inflammatory mechanisms of PDA@Exo in chondrocytes and its capacity to decelerate osteoarthritis progression, as inferred from RNA sequencing and KEGG GO analysis, Western blot assays were executed, targeting the phosphorylation profiles of essential proteins in the PI3K‐AKT‐mTOR signaling pathway. Figures 5h and Figure S19 (Supporting Information) demonstrate that PI3K, AKT, and mTOR phosphorylation levels are significantly elevated in the PDA, Exo, and PDA@Exo treatment groups. The PI3K‐AKT‐mTOR pathway, crucial for cell growth, survival, and metabolic regulation, showed an upregulation in response to IL‐1β stimulation, with marked increases in the phosphorylation of PI3K, AKT, and mTOR. PDA@Exo treatment moderated phosphorylation levels, indicating its regulatory impact on the pivotal PI3K‐AKT‐mTOR pathway and related cellular functions in chondrocytes, aligning with observed enhancements in chondrocyte proliferation, matrix synthesis, and cellular defense mechanisms. n conclusion, our research reveals that PDA@Exo adjusts the chondrocyte transcriptome to facilitate functional recovery, activate antioxidant enzymes, and reduce inflammation. These outcomes corroborate the theory that PDA@Exo reduces intracellular ROS and inflammation in cartilage, thus maintaining chondrocyte structural and functional integrity, and presenting a viable strategy for osteoarthritis treatment.

### 2.6 In Vivo Therapeutic Effect and Efficacy of PDA@Exo MN Patch on OA

**Figure 6:**
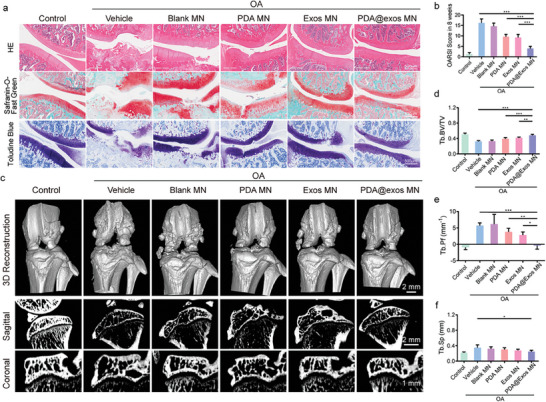
PDA@Exo attenuated OA progression in vivo. a) Representative images of H&E, TB, and SO‐FG staining in rat knee joints, and b) the corresponding OARSI scores after different treatments 8 weeks post‐surgery. Scale bars: 500 µm c) Representative 3D reconstruction images of differently treated rat knees at week 8 (Scale bars: 2 µm) and coronal micro‐CT images of the rat medial tibial plateau in different groups. (Scale bars: 1 µm); d–f) Quantitative analysis of BV/TV and increased Tb. Sp conducted by micro‐CT in 8 weeks post‐surgery. All data are shown as the mean ± standard deviation (SD). *p < 0.05, **p < 0.01, ***p < 0.001.

To assess the efficacy of PDA@Exo MN in treating rat knee osteoarthritis (OA), a model was established via anterior cruciate ligament transection (ACLT) and followed by intra‐articular administrations of treatments including control, Blank MN, PDA MN, Exo MN, and PDA@Exo MN, starting four weeks post‐surgery, every three days for a duration of four weeks. At eight weeks post‐treatment, the rats were euthanized for their knee joints to be analyzed with hematoxylin and eosin (H&E), safranin‐O/fast green (SO‐FG) staining and toluidine blue (TB), providing insight into the therapeutic impacts of the various treatments. Consequently, OA knees exhibited pronounced cartilage degradation and subchondral bone lesions, whereas PDA@Exo MN treatment preserved the integrity of both cartilage surfaces and subchondral bone. This protective effect on OA was further validated by OA Research Society International (OARSI) scores (**Figure** **6**a,b). Micro‐computed tomography (micro‐CT) analyses, including 3D reconstructions of the medial tibial plateau, illustrated typical OA manifestations like osteophyte formation and joint deformity in control OA knees of ACLT rats, which were absent in PDA@Exo MN‐treated groups (Figure 6c, upper row). Sagital and coronal micro‐CT images showed severe bone resorption and sparse trabeculae in control OA rats, whereas PDA@Exo MN treatment effectively mitigated these changes, improving trabecular bone volume and reducing trabecular separation as confirmed by micro‐CT quantitative analysis (Figure 6d–f).

### 2.7 In Vitro and In Vivo Biocompatibility Tests

After demonstrating the effects of PDA@Exo in vitro, further investigation in OA animal models is crucial to understand its potential in vivo. Animal models allow for evaluation of pharmacokinetics, biodistribution, efficacy, safety, and mechanisms of action of PDA@Exo in a more physiologically relevant context. HA has good degradation characteristics and HAMA has been proved to degrade in the presence of hyaluronidase and the result showed the MN dissolving progressively from 0 to 10 min, confirming their quick biodegradability and biosafety profile (Figure S20, Supporting Information).^[^ ^27^ ^]^ Histopathological evaluations of key organs, including heart, liver, spleen, lung, and kidney, in rats treated with PDA@Exo MN revealed no morphological or pathological changes, highlighting the treatment's non‐toxic nature (Figure S21, Supporting Information). Furthermore, histological assessments, including HE staining at microneedle application sites, confirmed an absence of inflammatory or pathological responses, with no erythema, edema, or cellular infiltration observed. Routine blood tests from the PDA@Exo MN‐treated animals revealed stable hematological parameters, with serum biochemical assays corroborating these findings. Liver and kidney function indicators, such as ALT, AST, and creatinine, remained within normal limits, indicating the absence of hepatic or renal toxicity due to the PDA@Exo MN treatment (Figure S22, Supporting Information). In conclusion, PDA@Exo MNs displayed exceptional biocompatibility and safety, as evidenced by their lack of toxicological impact on major organ functions, confirmed through histopathological and biochemical analyses. This foundational safety, coupled with their precise drug delivery, establishes these microneedles as a promising and effective method for osteoarthritis treatment.

## 3 Conclusion

**Scheme 1:**
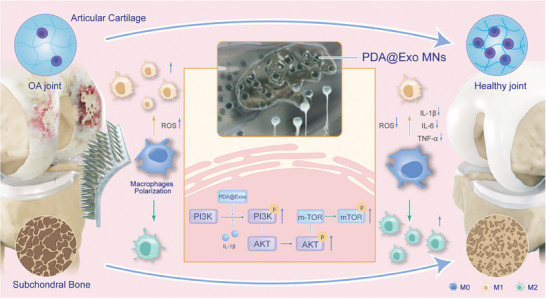
Schematic depicting PDA@Exo MN‐mediated modulation of cartilage degradation inhibition, osteogenesis enhancement, and macrophage polarization via the PI3K‐AKT‐mTOR signaling pathway.

In conclusion, our comprehensive studies affirm that PDA@Exo MNs serve as a potent intervention for osteoarthritis, providing dual benefits by reducing intracellular ROS and promoting osteogenesis while simultaneously preserving cartilage and promoting an M2 reparative macrophage phenotype. This therapeutic efficacy is supported by histological and micro‐CT analyses that show significant reduction of cartilage and bone damage in osteoarthritic models. Further pharmacokinetic assessments underscore the biocompatibility and safety profile of PDA@Exo MNs, positioning them as a promising, multifunctional approach to osteoarthritis treatment that concurrently addresses the disease's inflammatory and structural deteriorations, as depicted in **Scheme** **1**.

## 4 Experimental Section

### Statistical Analysis

Data were presented as mean ± SD and analyzed using GraphPad Prism software. Multiple comparisons among more than two groups were analyzed using multiple‐factorial ANOVA to determine statistical significance, indicated by *p < 0.05, **p < 0.01, ***p < 0.001. Student's t‐test or Welch's t‐test was used for two‐group comparisons, based on variance homogeneity.

## Conflict of Interest

The authors declare no conflict of interest.

## Supporting information

Supporting Information

## Data Availability

The data that support the findings of this study are available on request from the corresponding author. The data are not publicly available due to privacy or ethical restrictions.
