# Supplementary material for: Microneedle‐Delivered PDA@Exo for Multifaceted Osteoarthritis Treatment via PI3K‐Akt‐mTOR Pathway
Source: Adv Sci (Weinh). 2024 Aug 29;11(42):2406942. doi: 10.1002/advs.202406942 (PMC11558126; doi:10.1002/advs.202406942)
Supplement: Supplementary file 1 — Supporting Information [file ADVS-11-2406942-s001.pdf]

ADVANCED SCIENCE
Open Access

Supporting Information

for *Adv. Sci.*, DOI 10.1002/advs.202406942

Microneedle-Delivered PDA@Exo for Multifaceted Osteoarthritis Treatment via PI3K-Akt-mTOR Pathway

Zihua Li, Hengli Lu, Limin Fan, Xiaoyi Ma, Zhengwei Duan, Yiwei Zhang, Yuesong Fu, Sen Wang, Yonghao Guan, Dong Yang, Qingjing Chen, Tianyang Xu* and Yunfeng Yang**

Supplementary Information

Microneedle-Delivered PDA@Exo for Multifaceted Osteoarthritis Treatment via PI3K-AKT Pathway

Zihua Li[†], Hengli Lu[†], Limin Fan[†], Xiaoyi Ma, Zhengwei Duan, Yiwei Zhang, Yuesong Fu, Sen Wang, Yonghao Guan, Dong Yang, Qingjing Chen Tianyang Xu[*], Yunfeng Yang[*]*

Prof. Y.Yang
Department of Orthopaedics, Ruijin Hospital, Shanghai Jiao Tong University School of Medicine, Shanghai 200025, P. R. China.
E-mail: dr_yangyf123@163.com

Dr. Z. Li, H. Lu, Z. Duan, Y. Zhang, Y. Fu, S. Wang, Y. Guan, D. Yang, Prof. T. Xu
Department of Orthopedics, Shanghai Tenth People's Hospital, School of Medicine, Tongji University, Shanghai 200072, P. R. China.
E-mail: xutianyangty@163.com

Dr. L. Fan, X. Ma
School of Medicine, Tongji University, Shanghai 200092, P. R. China.

Dr. Q. Chen
Southern Medical University, Guangzhou, 510515, P. R. China.
E-mail:hingching@126.com

A. Experimental Section

Materials and Methods

Materials

HA (10 kDa) and 150 kDa Methacrylated hyaluronic acid (HAMA) were obtained from Dalian Meilun Biotechnology Co., Ltd. (China) and EngineeringForLife company (Suzhou, China), respectively. 2-hydroxy-2-methylpropiophenone (HMPP) and dopamine hydrochloride were purchased from Sigma-Aldrich (Shanghai, China). DCFH-DA (2",7"-dichlorodihydrofluorescein diacetate) was purchased from MedChemExpress company (NJ, USA). Calcein AM, PI, Hoechst 33 342, BeyoClick EdU Cell Proliferation Kit, and Cell Counting Kit-8 (CCK-8) were obtained from Beyotime (China). Additionally, we obtained DiD Perchlorate (The 1,1'-dioctadecyl-3,3,3',3'tetramethylindodicarbocyanine,4-chlorobenzenesulfonate salt) and DiO perchlorate (3,3-dioctadecyloxacarbocyanine perchlorate) from Yeasen Biotechnology Co., Ltd. (Shanghai, China). ELISA kits were obtained from BioLegend Company (San Diego, CA, USA). Dulbecco's modified Eagle's medium (DMEM) and DMEM/F12, as well as penicillin-streptomycin (PS) and phosphate-buffered saline (PBS), were purchased from HyClone in Logan, UT, USA. Monoclonal antibodies CD9, Alix, TSG101, and Calnexin were obtained from Abcam in Cambridge, UK. Cell counting kit-8 (CCK-8), calcein-acetoxymethyl ester (calcein-AM), propidium iodide (PI), 2', 7'-dichlorodihydrofluorescein diacetate (DCFH-DA), 4', 6'-diamidino-2-phenylindole (DAPI), superoxide dismutase (SOD) assay kit, lipid peroxidation malondialdehyde (MDA) assay kit, mitochondrial membrane potential assay kit, Trizol reagent, adenosine triphosphate (ATP) assay kit, dexamethasone, 4% paraformaldehyde fix solution,

bovine serum albumin (BSA), and alizarin red S were obtained from Shanghai Beyotime Biotechnology Co., Ltd. 0.025% collagenase type II was purchased from Thermo Fisher Scientific. Lipopolysaccharide (LPS), β-sodium glycerophosphate, and L-ascorbic acid were obtained from Sigma-Aldrich. Hyaluronic acid (HA) was purchased from Shanghai Aladdin Biochemical Technology Co., Ltd., and dissolved with normal saline into 10 mg/mL before usage.

PDA and PDA NPs synthesis

PDA was synthesized by mixing 0 mM 3-hydroxytyramine hydrochloride with 1 M NaOH for 5 hours at room temperature, then centrifuged, washed, and stored at 4°C. PDA NPs were prepared via the Stöber method, involving NH4OH, ethanol, and water, then adding dopamine hydrochloride, stirring for a day, washing, and freeze-drying. The synthesized NPs underwent cytotoxicity evaluation using a CCK-8 assay and Calcein-AM/PI staining, with their morphology and size distribution assessed by TEM and Image-J analysis.

Preparation and Characterization of MSC-Exos

The ultracentrifugation method was used to isolate exosomes from the medium as previously described. Briefly, the exosome-free serum medium was collected and subjected to multiple rounds of centrifugation at increasing speeds (300 × g for 10 min, 3000 × g for 30 min, 10,000 × g for 60 min, and 100,000 × g for 70 min at 4°C). The resulting pellet was resuspended in PBS, filtered through a 0.22-μm filter, and centrifuged again at 100,000 × g for 70 min at 4°C. The purified exosomes were characterized by TEM and NTA to determine size and concentration. The protein concentration of MSCs-Exo was analyzed using the Pierce BCA Protein Assay Kit.

Western blot analysis was performed to identify exosomal markers CD9, Alix, TSG101, and Calnexin.

Fabrication of MN Patch

Concisely, Precise quantities of low molecular weight hyaluronic acid, high molecular weight hyaluronic acid, and hydroxypropyl-β-cyclodextrin are measured and combined with distilled water under ultrasonic agitation, resulting in a mixture with mass fractions of 9.33%, 1.33%, and 5.33% for each component. Similarly, the PDA@Exo MN solution is prepared by mixing specific masses of the above components with PDA@Exo nanoparticle solution. The same approach is taken for FITC and methylene blue microneedle solutions using specific masses of these components mixed with 10ml 50ug/ml FITC. Subsequently, the MNs are fabricated using micro-molding. The pre-mixed solution is poured into a Polydimethylsiloxane (PDMS) mold, subjected to vacuum treatment for complete filling, and followed by adding more solution for backing. After 3 minutes of vacuum treatment repeated three times, the mold is dried for 24 hours at 25°C to complete the microneedle patch preparation.

Characterization

Surface morphology of the MNs was characterized by both scanning electronic microscopy (SEM) and CLSM. For SEM visualization, the MNs were coated with a thin layer of carbon and visualized using a TESCAN MIRA LMS (Brno, Czech Republic). For visualization by CLSM, the MNs were scanned with a step speed of 5 μm/step by using a 10× Plan Apo objective. Utilizing micromanipulation, the mechanical properties of individual microneedles, including their rupture behavior, were comprehensively evaluated according to a well-established protocol.

Specifically, mechanical characterization involved both tensile and compression tests. Tensile testing applied static load along the sample's longitudinal axis under controlled conditions. Transmission electron microscopy (TEM) and high-resolution transmission electron microscopy (HRTEM) was conducted on a Tecnai F30 (FEI, Netherlands) microscope. The scanning electron microscopy (SEM) images of BPNSs were obtained by an S4800 (Hitachi, Japan) field emission scanning electron microscope. Atomic force microscopy (AFM) was conducted to determine the thickness of BPNSs through a Dimension Icon (Bruker, Germany) atomic force microscope. The dynamic light scattering (DLS) measurement was applied to characterize the average particle size of BPNSs using a Zetasizer Nano ZS90 (Malvern, Britain). Zeta potential was detected in water (pH 7.0) through a Mastersizer 3000 (Malvern, Britain) laser diffraction particle size analyzer. UV-vis spectra were detected with a UV-1900i UV-vis spectrophotometer (Shimadzu, Japan). Fourier transform infrared (FTIR) spectra of BPNSs were performed on a Nicolet iS10 FTIR spectrometer (Thermo Scientific, USA). Raman spectra were performed using an inVia Raman Microscope (Renishaw, Britain). Electron spin resonance (ESR) spectra were conducted on an A300 ESR spectrophotometer (Bruker, Germany). Confocal images were obtained by confocal laser scanning microscopy observation (LSM 710; Carl Zeiss, Germany). The knees were scanned using a micro-computed tomography (micro-CT) equipment (Nemo NMC-100, PINGSENG Healthcare Inc., China). Load increased until specimen fracture, collecting stress-strain data. Stress (σ) was calculated ($\sigma = F/A$), and strain (ε) as the ratio of length change (Δl) to original length ($l0$) ($\varepsilon = \Delta l/l0$). The resulting curve revealed mechanical parameters like tensile strength, ultimate tensile stress, yield strength, and modulus of elasticity. Compression experiments employed a P/20Rmm cylindrical indenter with 0.1 mm/s testing speed. Samples had standardized dimensions (2000 mm thickness, 100 mm^2 surface area)[1].

Cell isolation and culture

Primary chondrocytes and bone marrow mesenchymal stem cells (BMSCs) were harvested from Sprague-Dawley rats for cellular studies. The chondrocytes were isolated by dissecting the hind limbs, meticulously removing the skin and muscle to expose knee joint cartilage, which was then minced and subjected to enzymatic dissociation using 0.25% trypsin-EDTA solution for 45 minutes at 37°C. The enzymatic action was neutralized with complete culture medium, and the resultant cell suspension was centrifuged to discard the supernatant. The cell pellet received further treatment with Type II collagenase overnight at 37°C to ensure complete digestion. Following centrifugation and PBS washes, chondrocytes were resuspended in high glucose DMEM supplemented with 10% fetal bovine serum and 1% penicillin/streptomycin and incubated in a CO_2 incubator. Simultaneously, BMSCs were obtained by sacrificing the rats and extracting the marrow from femurs and tibias. After removing the bone ends, the marrow cavities were flushed with complete medium until the bones appeared pale, a process which also served to collect the marrow cells. Media was refreshed every 24 hours to promote the growth of pure BMSC cultures, which were then maintained in low glucose DMEM with similar supplements as the chondrocytes. RAW264.7 cells were sourced from the Cell Bank of the Chinese Academy of Sciences. RAW 246.7 cells were grown in high glucose DMEM. All cultures included 10% FBS and were kept at 37 °C in a humidified 5% CO_2 atmosphere. Antibiotics (100 U/ml penicillin G and 100 μg/ml streptomycin sulfate) were consistently present to prevent contamination. The RAW264.7 cell line was stimulated with 50 ng/mL LPS 24h for macrophage differentiation.

In Vitro Cell Experiments

Primary chondrocytes from SD rats were cultured in DMEM/F12 with 10% FBS until 80% confluence, then treated with Control, PDA, Exo, and PDA@Exo for further analysis. To assess the cell viability and biocompatibility of biomaterials, we employed a CCK-8 assay and Calcein-AM/Propidium Iodide (PI)staining, respectively. Calcein-AM/PI staining allowed visual assessment of live and dead cells after treatment with control, PDA, Exo, and PDA@Exo under H_2O_2-induced oxidative stress. To assess the cell viability and biocompatibility of biomaterials, we employed a CCK-8 assay and Calcein-AM/Propidium Iodide (PI)staining, respectively.

Subsequently, they were incubated with the ROS-sensitive dye DCFH-DA for 30 minutes. Additionally, the ATP content of the chondrocytes was measured using an ATP assay kit following the manufacturer's instructions. The JC-1 fluorescence was observed using a fluorescence microscope, and the results were analyzed quantitatively using ImageJ software. Cellular SOD activity was further determined in H_2O_2-induced oxidative stress cells using a total SOD assay kit. To evaluate the lipid peroxidation levels of differently treated chondrocytes, a lipid peroxidation MDA assay kit was applied. To assess the intracellular oxidative stress, western blot analysis was performed for key proteins including Caspase 3, cleaved Caspase 3, Caspase 9, Bax, and Bcl-2. Additionally, RT-PCR analysis was used to evaluate the expression levels of essential antioxidant enzymes (SOD3, GPX, CAT), inflammatory markers (iNOS, COX-2), and cartilage matrix anabolic (COL2A1, ACAN, SOX9) and catabolic markers (ADAMTS5, ADAMTS1, MMP13). The CLSM images were obtained using an Olympus FV3000 confocal microscope. The osteogenic differentiation medium for BMSCs treated with Control, PDA, Exo, and PDA@Exo was prepared as follows: DMEM medium was supplemented with β-sodium glycerophosphate (10 mM), L-ascorbic acid (50 μg/mL), and dexamethasone (10 nM). BMSCs were then cultured in the osteogenic differentiation medium for 21 days, and the formation of calcium nodules during the

osteogenic process was observed by alizarin red S staining, following the manufacturer's instructions. The expression of osteogenesis-associated genes, including OPN, RUNX2, and BMP2, in BMSCs was further analyzed using western blot.

Macrophages Polarization

RAW 264.7 cells were seeded at 10^5 cells per well in 12-well plates across five groups; four were treated with 50 ng/mL LPS to induce an inflammatory response and subsequently administered PDA, Exo, PDA@Exo, or vehicle, while the control group received no additions. Following a 24-hour incubation period, RAW 264.7 cells underwent immunostaining for CD206 and iNOS to ascertain macrophage polarization. Fluorescently labeled antibodies targeting CD206 and iNOS facilitated the cells' visualization with high-resolution confocal microscopy using an Olympus FV3000 system. Concurrently, the secretion levels of pro-inflammatory cytokines IL-6, TNF-α, and IL-1β in the collected supernatants were assessed using specific ELISA assays.

RT-PCR

Cells were collected and treated with a Trizol reagent to extract total RNA. Then the reverse transcription was conducted using RevertAid First Strand cDNA Synthesis Kit to obtain cDNA. The resulting cDNA was then used for RT-PCR with corresponding primers and FastStart Universal SYBR Green Master (Rox) (Roche, Switzerland) in a Real-Time PCR System (StepOnePlus, ABI, USA). The detailed information on the primers was listed in **Table S1**. Quantitative expression analysis was carried out using the ABI 7500 real-time PCR system (Applied Biosystems, CA, USA). The relative expression levels of the target mRNAs were evaluated using the $2^{-\Delta\Delta Ct}$ method.

Western blotting

Cells or exosomes were lysed with RIPA buffer containing in a complete protease and phosphatase inhibitor. Total proteins were separated by SDS-PAGE gel and transferred onto polyvinylidene difluoride (PVDF) membranes (Millipore, USA). Then blocked in 5% skim milk for 1 h, membranes were incubated with the indicated primary antibodies at 4 °C overnight, including anti-Alix, anti-TSG101, anti-CD9 and anti-Calnexin (Abcam, #ab275377, #ab125011, #ab263019, # ab 133615, 1:1000), anti-β-actin (Abcam, #ab92486, 1:1000), anti-Caspase 3 (Abcam, #ab13847, 1:1000), anti-cleaved Caspase 3 (Abcam, #ab2302, 1:1000), anti-Caspase 9 (Abcam, # ab222231, 1:1000), anti-Bax (Abcam, #ab32503, 1:1000), anti-Bcl-2 (Abcam, #ab32124, 1:1000), anti-MMP9 (Abcam, #ab38898, 1:1000), anti-MMP13 (Abcam, #ab39012, 1:1000), anti-RUNX2 (Abcam, #ab76956, 1:1000), anti-SOX9 (Abcam, #ab185966, 1:1000), anti-OPN (Abcam, #ab283656, 1:1000), anti-BMP2 (Abcam, #ab284387, 1:1000), and anti-COL2 (Abcam, #ab34712, 1:1000), anti-CD206 (Abcam, #ab64693, 1:1000), anti-Arg1 (Abcam, #ab91279, 1:1000), anti-iNOS (Abcam, #ab15323, 1:1000), anti- PI 3 Kinase p85 alpha (Abcam, #ab191606, 1:1000), anti- Akt (CST, #4691, 1:1000), anti-Phospho-Akt (Thr308) (CST, #4056, 1:1000), anti-mTOR (Abcam, ab134903, 1:1000), anti-Phospho-m-TOR (Abcam, ab109268, 1:1000). Further incubation with secondary antibodies was performed, and target proteins were detected using the enhanced chemiluminescence system (GE Health Care, Piscataway, NJ), followed by band analysis with Image J software to quantify protein expression as the ratio of the peak area of target proteins to that of the endogenous controls, β-actin or GAPDH.

RNA-sequencing of chondrocytes

Chondrocytes were categorized into three groups for culture and subsequent treatment. The OA model group was induced with IL-1β at a concentration of 10 ng/mL to mimic OA conditions, conducted in triplicate, to facilitate comparison with the non-treatment control group. In contrast, the PDA@Exo group received a combination of IL-1β (10 ng/mL) and PDA@Exo, with three replicates. After 24 hours of incubation, the cells were collected and treated with Trizol reagent to extract total RNA for RNA sequencing, which was performed with a BGI sequencing platform. Gene expression was evaluated using the FPKM method, and differential expression analysis was conducted using the DESeq algorithm with significant analysis based on the P-value and false discovery rate (FDR). Differentially expressed genes were identified using the filtering threshold of FDR < 0.05, $log_2FC > 1$, or $log_2FC < -1$. GO analysis was performed using the R-package clusterprofiler, and Fisher's exact test was used for significant analysis of enriched GO categories. Pathway analysis was conducted on differentially expressed genes to identify significantly influenced pathways in the PDA@Exo treatment group, based on the KEGG database using the R-package clusterprofiler. Fisher's exact test was used to identify significantly enriched pathways with the threshold of significance defined by the P value. A GO Chord plot was used to illustrate the relationship between representative GO terms and interested DEGs. The circular heatmap of interested differentially expressed genes, the classification summary plot of KEGG pathway analysis, and the GO Chord plot were plotted using an online platform for data analysis and visualization (http://www.bioinformatics.com.cn)[2].

Construction of ACLT-induced OA models

Sprague-Dawley (SD) rats weighing 180-220 g were obtained from Shanghai Slac Laboratory Animal Co., Ltd. maintained in the Tongji University. The experimental protocol was approved

by the Animal Care and Use Committee of Tongji University. The therapeutic effect of PDA@Exo MN on osteoarthritis (OA) in vivo was evaluated by performing anterior cruciate ligament transection (ACLT) on SD rats to create a knee OA animal model[3]. The animals were randomly assigned to one of six groups: control, OA + (Blank MN, PDA MN, Exo MN, or PDA@Exo MN). Under appropriate anesthesia using isoflurane, the anterior cruciate ligament in the right leg of the animal was exposed and transected under direct vision in the control and treatment groups. Different MN were administered 4 weeks after the operation, once every 3 days, for 4 weeks. The rats were sacrificed 8 weeks after ACLT surgery for further experiments.

Histological assessment

Following the scheduled treatment, knee joints of the OA rats that underwent anterior cruciate ligament transection (ACLT) were collected and fixed in 4% paraformaldehyde solution for 3 days. Hematoxylin and eosin (H&E), toluidine blue (TB), and safranin-O/fast green (SO-FG) staining was performed to enable histological evaluation. The quantitative analysis of histological changes in cartilage was conducted using the Osteoarthritis Research Society International (OARSI) scores. The dissected knee joints were fixed in a 4% paraformaldehyde solution for three days. Subsequently, the knees were scanned using a micro-CT equipment (Nemo NMC-100, PINGSENG Healthcare Inc., China), and the scanning profiles were analyzed using Avatar software 1.6.6 to generate three-dimensional reconstruction images of the knee joints. The trabecular bone volume per total volume (BV/TV) and trabecular separation (Tb. Sp) results were also obtained through this analysis.

Evaluation of PDA@Exo MN Dissolution Kinetics and Safety Profile in Biological Systems

Methylene blue staining facilitated nucleic acid visualization by binding to the molecules, highlighting cell nuclei in blue. The process involved: gently inserting microneedle patches into rat skin for 2 hours; carefully collecting and fixing tissue samples with 4% paraformaldehyde; promptly cryopreserving tissue blocks to preserve enzymatic activity; applying OCT embedding gel and cooling specimens on a holder; generating tissue sections (5-10 μm) with a microtome; proceeding through fixation, washing, and antigen retrieval steps; drying and sealing sections on slides; finally, methodically preserving sections at 4°C. To investigate the dissolution kinetics of fabricated microneedles, MNs were submerged in distilled water at room temperature. A time-lapse camera was positioned to capture the dissolution process from 0 to 10 minutes.

On day 28 post-treatment, the heart, liver, spleen, lung, and kidney were excised from the mice and fixed in 10% buffered formalin. After embedding in paraffin, 3 μm-thick tissue sections were obtained and stained with hematoxylin and eosin (H&E) to assess possible toxicity to major organs and cytotoxicity to MN. Blood was collected before sacrificing the mice for routine and biochemical tests to evaluate systemic effects.

[1] G. Du, Z. Zhang, P. He, Z. Zhang, X. Sun, J Mech Behav Biomed Mater 2021, 117, 104384.
[2] Y. Benjamini, D. Drai, G. Elmer, N. Kafkafi, I. Golani, Behav Brain Res 2001, 125, 279; M. Ashburner, C. A. Ball, J. A. Blake, D. Botstein, H. Butler, J. M. Cherry, A. P. Davis, K. Dolinski, S. S. Dwight, J. T. Eppig, M. A. Harris, D. P. Hill, L. Issel-Tarver, A. Kasarskis, S. Lewis, J. C. Matese, J. E. Richardson, M. Ringwald, G. M. Rubin, G. Sherlock, Nat Genet 2000, 25, 25; G. Yu, L. G. Wang, Y. Han, Q. Y. He, Omics 2012, 16, 284.
[3] E. L. Kuyinu, G. Narayanan, L. S. Nair, C. T. Laurencin, J Orthop Surg Res 2016, 11, 19.

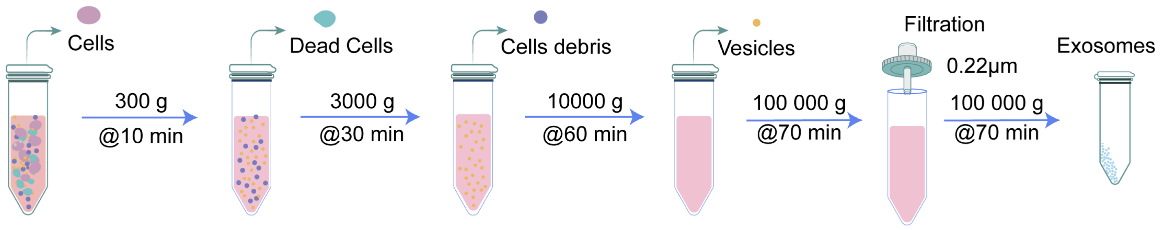

Figure S1: Schematic representation of the ultracentrifugation steps for isolating exosomes from cell culture supernatant.

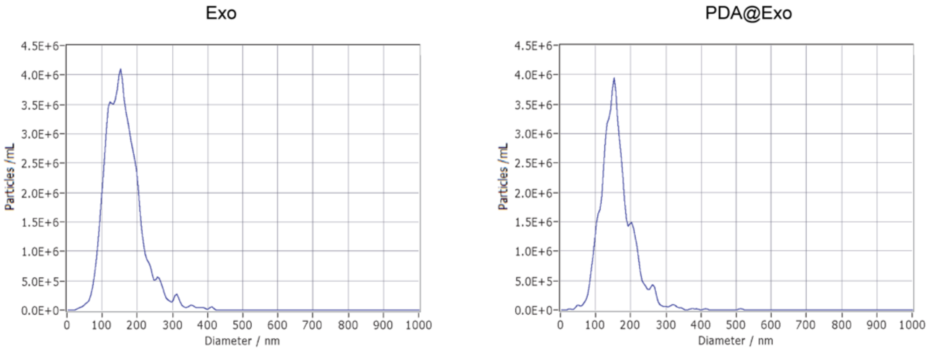

Figure S2: Nano tracking analysis of Exo and PDA@Exo.

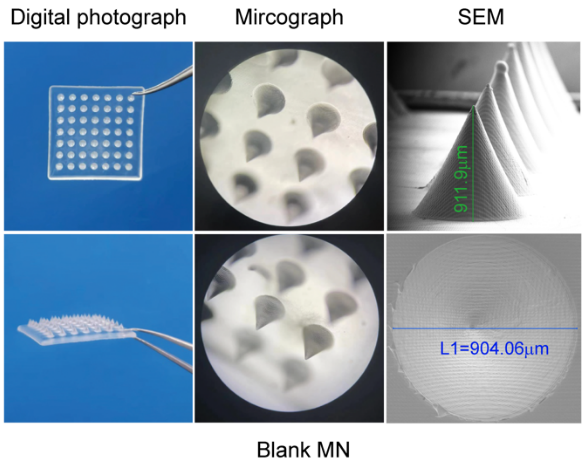

Figure S3: Morphological Characterization of blank showing digital photographs, light micrographs, and scanning electron microscopy (SEM) images of the MN, respectively.

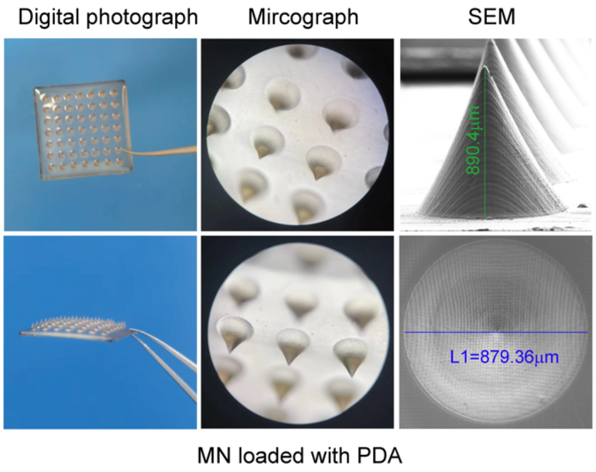

Figure S4: Morphological Characterization of PDA-loaded MNs showing digital photographs, light micrographs, and scanning electron microscopy (SEM) images of the MN, respectively.

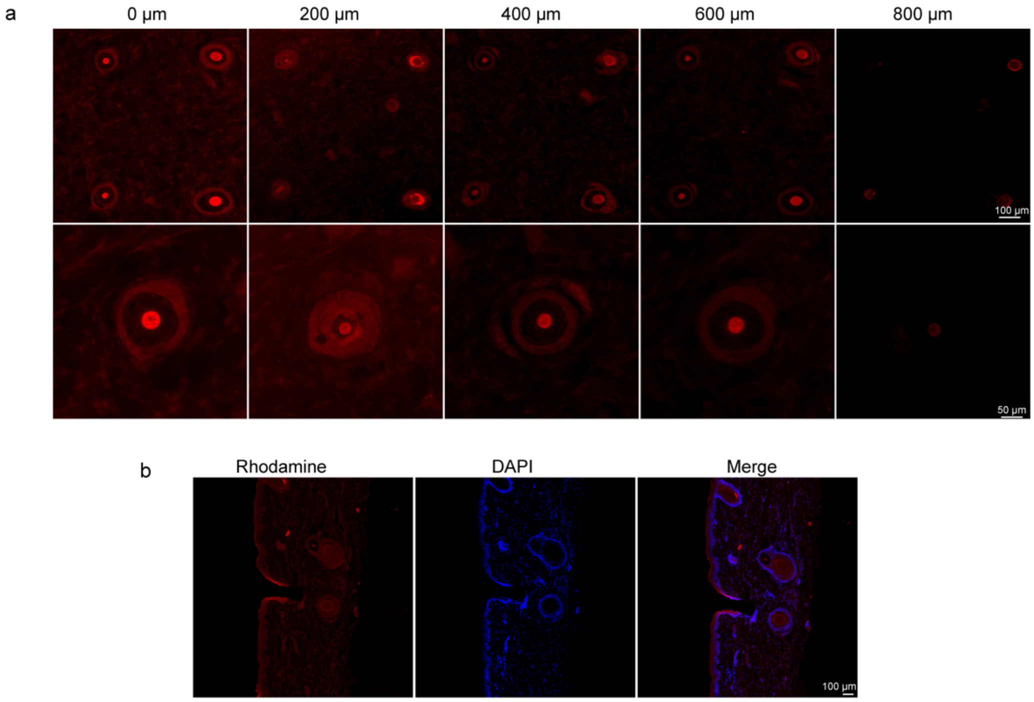

Figure S5: **(a)** CLSM images of (a) PDA@Exo-PKH26@MN array at varying depths: 0, 200, 400, 600, 800 μm. Scale bars: 100 μm and 50 μm. **(b)** Distribution of PDA@Exo-PKH26@MN within the skin layers. Scale bars: 100 μm.

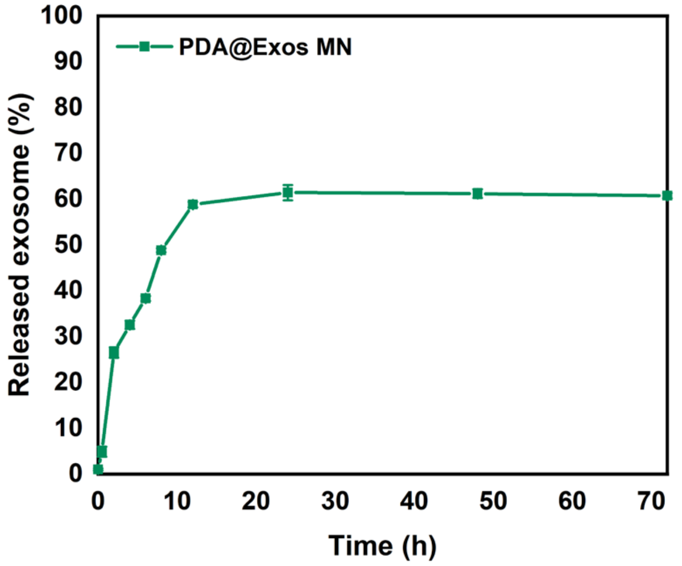

Figure S6: Release of Exos from microneedle with different formulations in vitro.

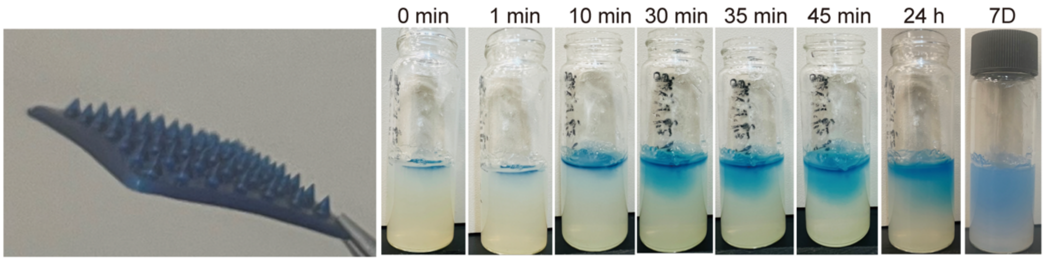

Figure S7: Time-course visualization of methylene blue-labeled MNs dissolving in an agarose gel.

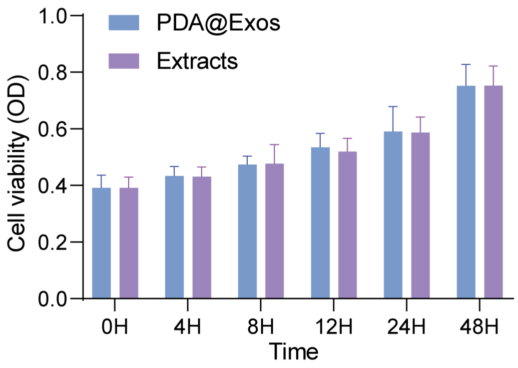

Figure S8: Chondrocytes viability assessed by CCK-8 assay after treatment with PDA@Exo and PDA@Exo MN extracts. All data are shown as the mean ± standard deviation (s. d.). *$P < 0.05$, **$P < 0.01$, ***$P < 0.001$.

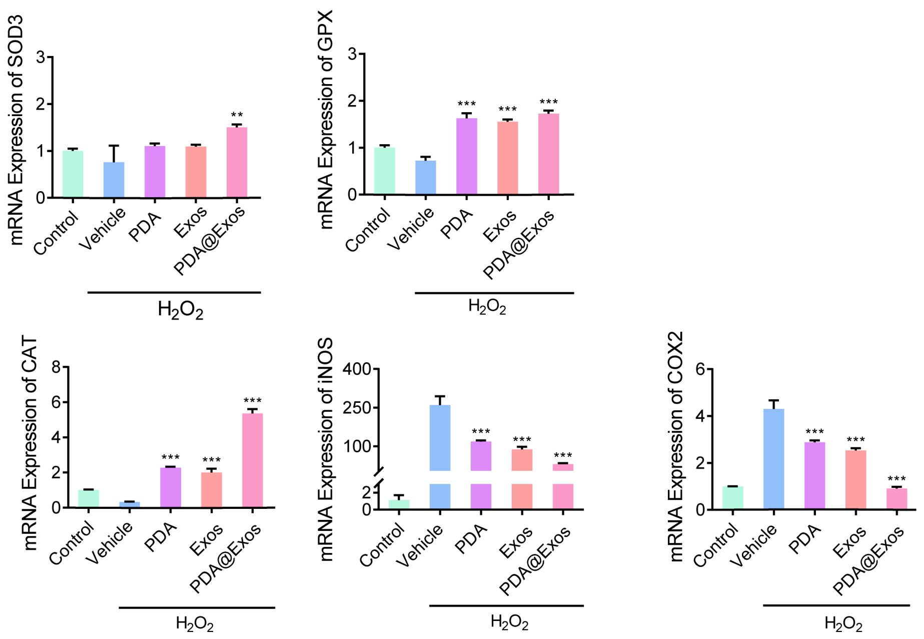

Figure S9: Quantitative analysis antioxidant enzymes (SOD3, GPX, and CAT) and inflammatory biomarkers (iNOS and COX2) was assessed by RT-PCR. All data are shown as the mean ± standard deviation (s. d.). *P < 0.05, **P < 0.01, ***P < 0.001.

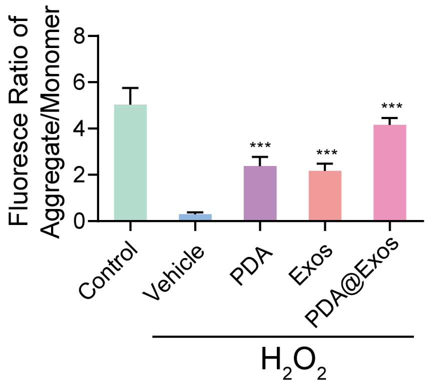

Figure S10: Quantitative fluorescence ratio analysis of JC-1 aggregates to monomers (A/M ratio). All data are shown as the mean ± standard deviation (s. d.). *$P < 0.05$, **$P < 0.01$, ***$P < 0.001$.

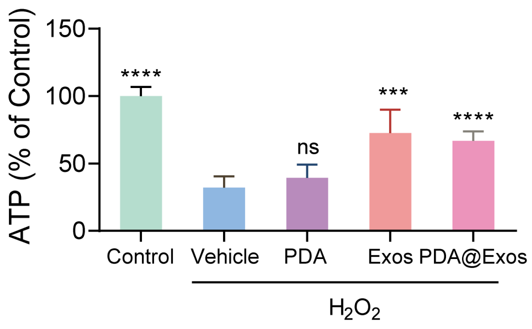

Figure S11: ATP content of chondrocytes treated with Control, PDA, Exo, and PDA@Exo under H_2O_2 different treatments. All data are shown as the mean ± standard deviation (s. d.). $*P < 0.05$, $**P < 0.01$, $***P < 0.001$, compared to H_2O_2-induced chondrocytes.

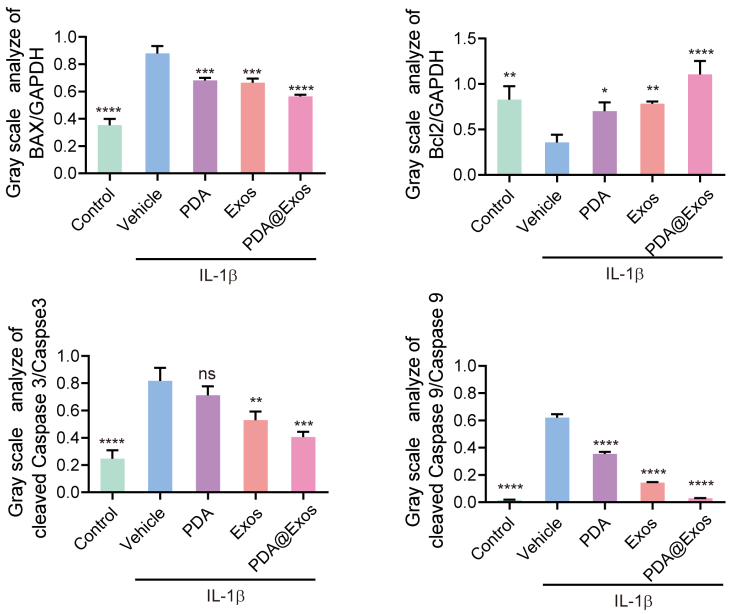

Figure S12: Quantification of Western blot analysis of apoptosis-related proteins in chondrocytes, including BAX, Bcl-2, cleaved Caspase-3/Caspase-3, cleaved Caspase-9/Caspase-9. All data are shown as the mean ± standard deviation (s. d.). $*P < 0.05$, $**P < 0.01$, $***P < 0.001$.

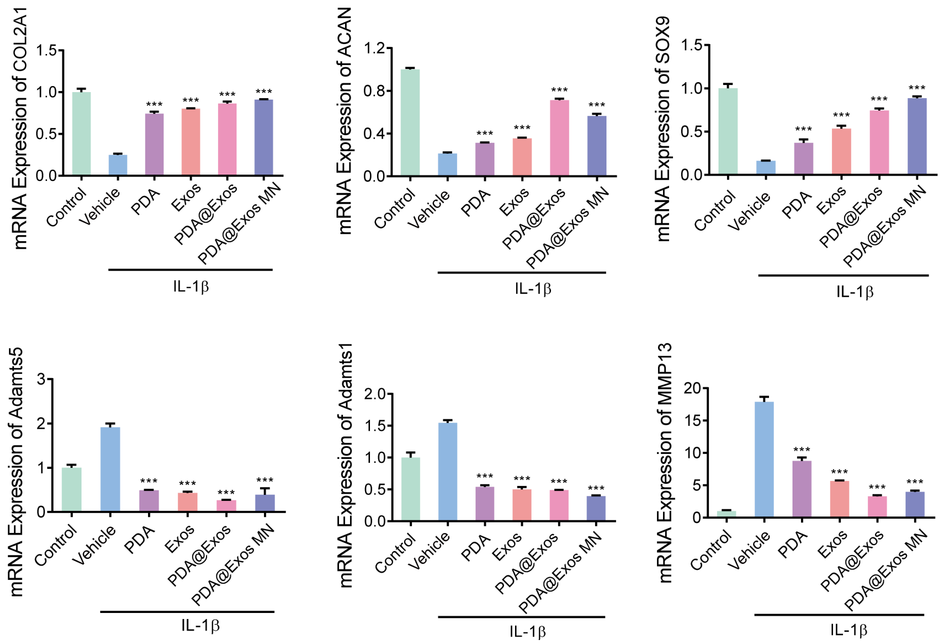

Figure S13: Quantitative analysis of mRNA expression levels corresponding to cartilage matrix anabolic factors (COL2A1, ACAN, SOX9) and catabolic enzymes (Adamts5, Adamts1, MMP13) in chondrocytes. All data are shown as the mean ± standard deviation (s. d.). *$P < 0.05$, **$P < 0.01$, ***$P < 0.001$.

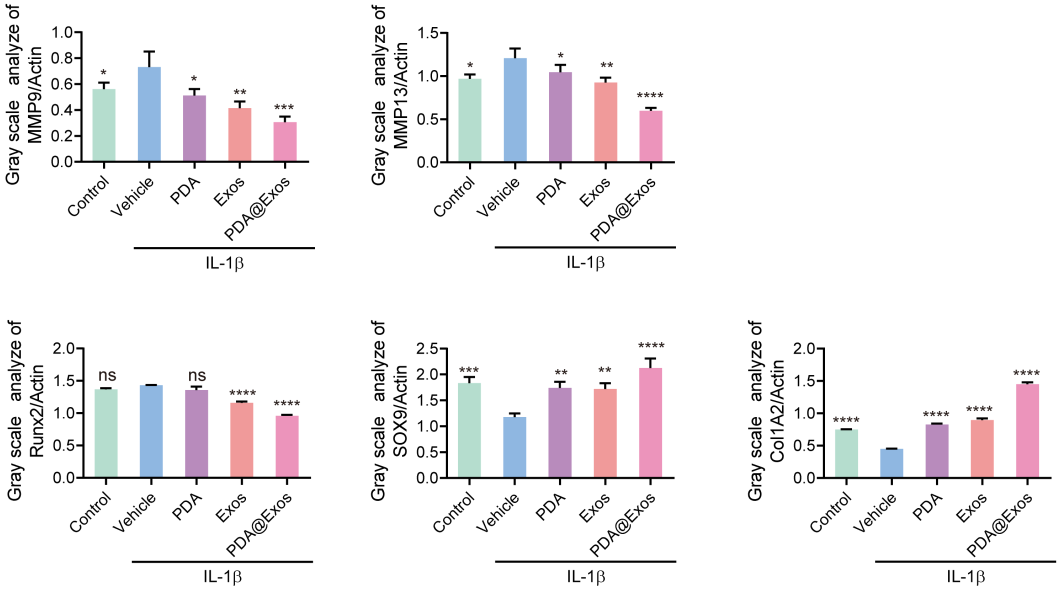

Figure S14: Quantification of Western blot analysis showing the expression levels of MMP9, MMP13, RUNX2, SOX9 and COL1A2 in chondrocytes. All data are shown as the mean ± standard deviation (s. d.). *$P < 0.05$, **$P < 0.01$, ***$P < 0.001$.

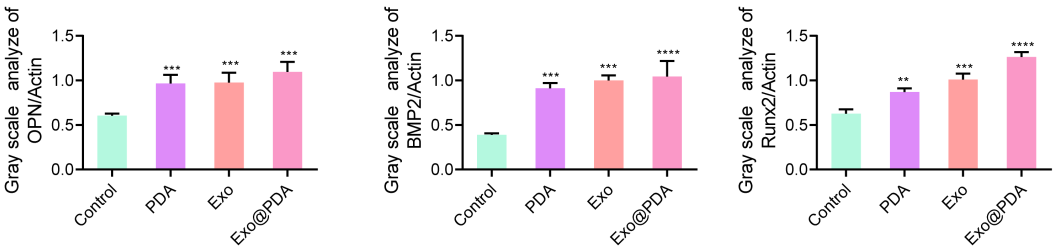

Figure S15: Quantitative analysis of Western blot for osteogenesis-related protein expression (OPN, RUNX-2, BMP-2) in BMSCs. All data are shown as the mean ± standard deviation (s. d.). *$P < 0.05$, **$P < 0.01$, ***$P < 0.001$.

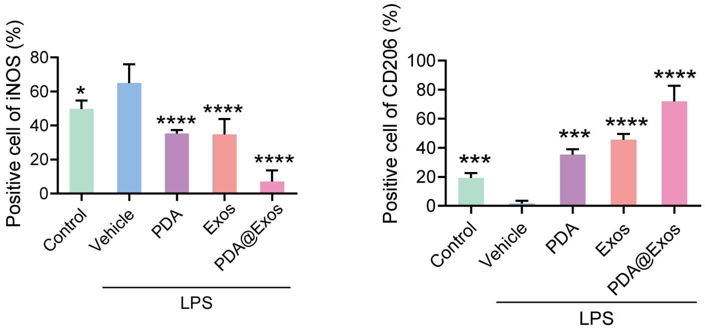

Figure S16: Fluorescence intensity analysis for the immunolabeling of iNOS and CD206 in RAW264.7 cells. All data are shown as the mean ± standard deviation (s. d.). $*P < 0.05$, $**P < 0.01$, $***P < 0.001$.

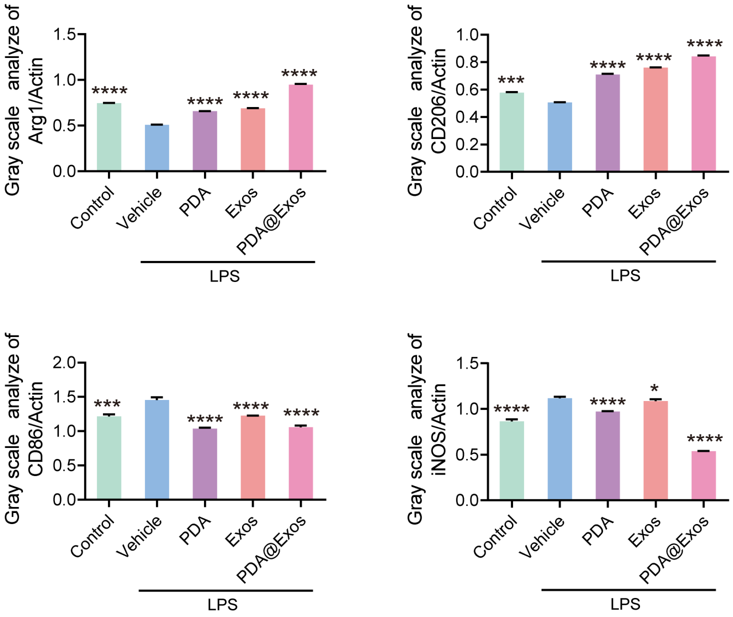

Figure S17: Quantification of Western blot analysis for macrophage polarization markers (Arg1, CD206, CD86, iNOS) in RAW264.7 cells. All data are shown as the mean ± standard deviation (s. d.). **P* < 0.05, ***P* < 0.01, ****P* < 0.001.

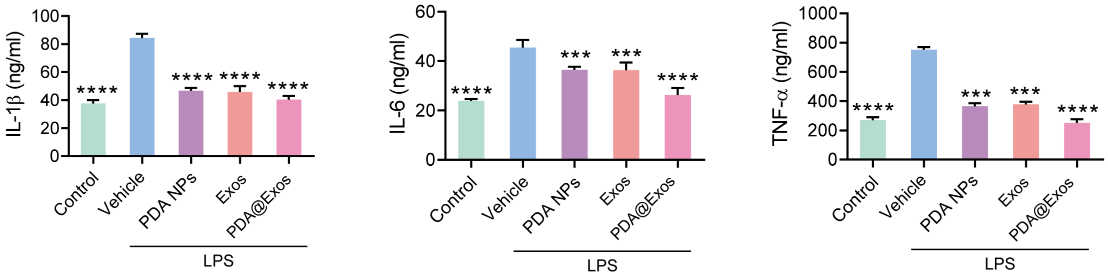

Figure S18. The anti-inflammatory ability of PDA@Exo in RAW264.7 cells stimulated with LPS was monitored using enzyme-linked immunosorbent assay (ELISA) of (a) IL-1β, (b) IL-6, and (c) TNF-α. All data are shown as the mean ± standard deviation (s. d.). $*P < 0.05$, $**P < 0.01$, $***P < 0.001$.

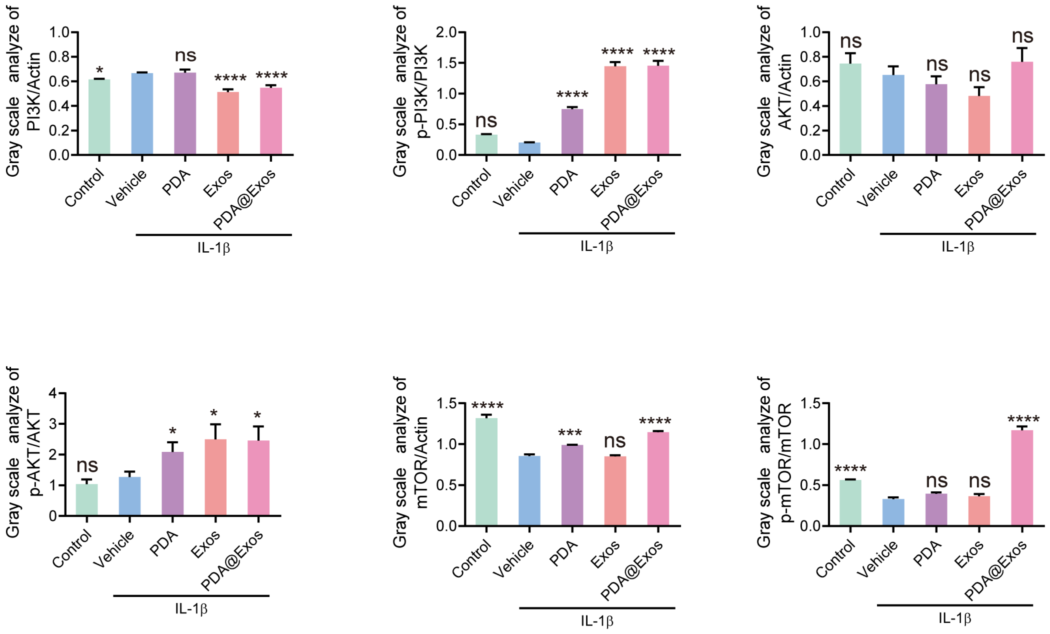

Figure S19: Quantification of Western blot analysis showing the expression levels of in PI3K/AKT/mTOR pathway (PI3K, p-PI3K, AKT, p-AKT, mTOR, p-mTOR) in chondrocytes. All data are shown as the mean ± standard deviation (s. d.). *$P < 0.05$, **$P < 0.01$, ***$P < 0.001$.

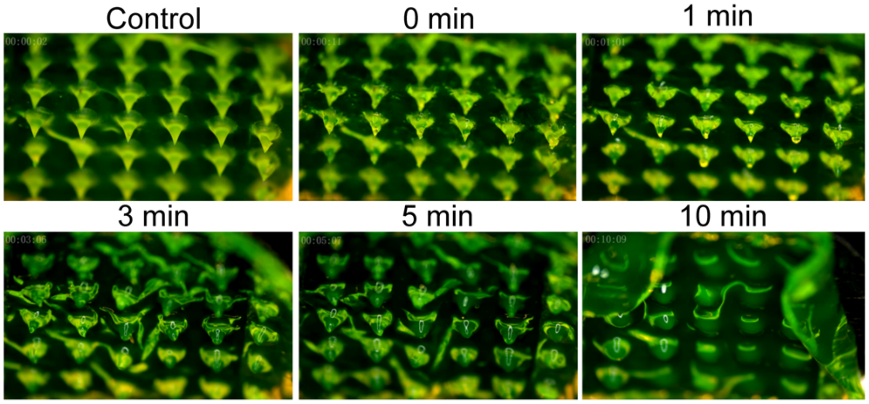

Figure S20: Time-lapse imaging of MN dissolution in water. The series captures the progressive disintegration of microneedles at various time points (0, 1, 3, 5, and 10 minutes) post-immersion.

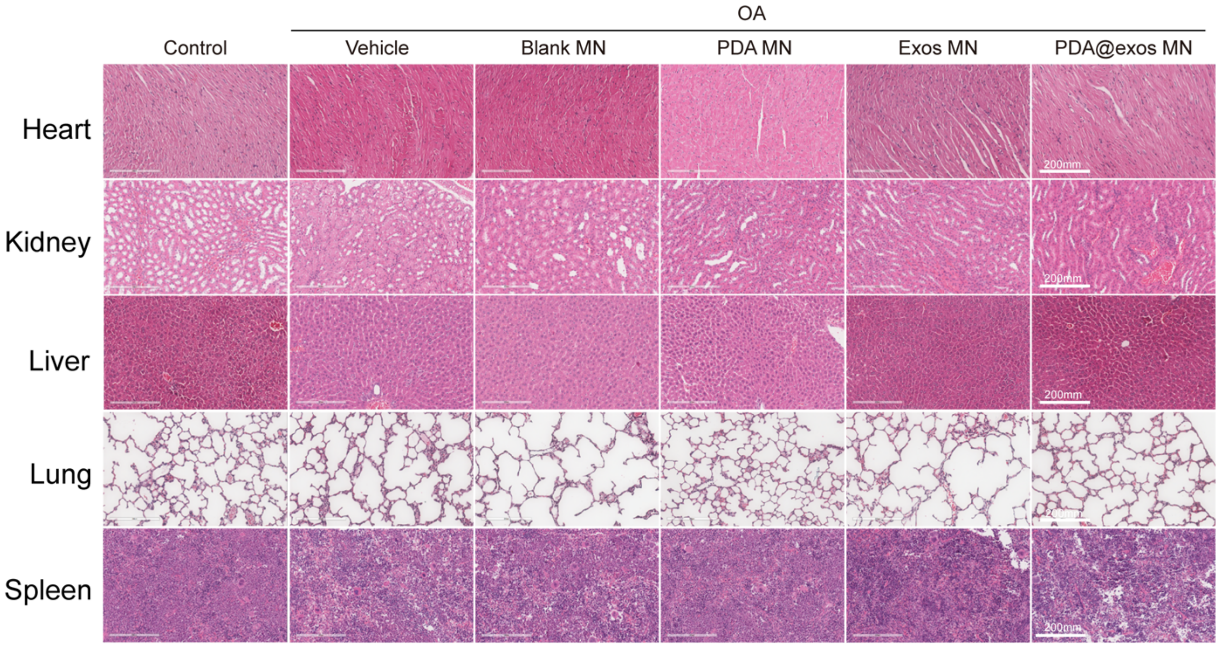

Figure S21. H&E-stained sections of rat heart, kidney, liver, lung, and spleen from Sham, OA, and OA groups treated with PDA MN, Exos MN, and PDA@Exos MN after a 4-week intervention

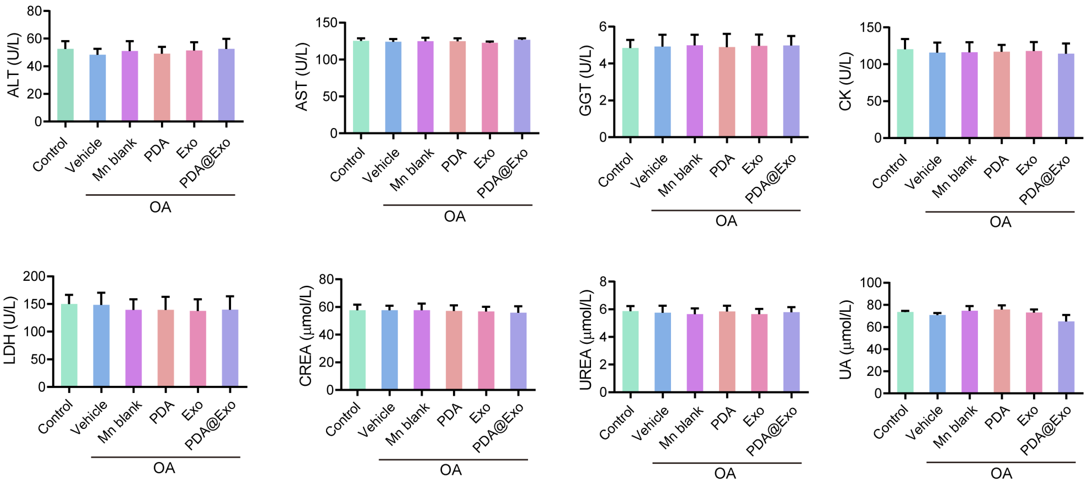

Figure S22. Biochemical analysis of serum markers indicating organ function in OA-induced rats treated with various interventions. The panels display levels of ALT, AST, GGT, CK, LDH, CREA, UREA, and UA, comparing the control group with groups treated with Vehicle, Mn blank, PDA, Exo, and PDA@Exo MN. All data are shown as the mean ± standard deviation (s. d.). **P* < 0.05, ***P* < 0.01, ****P* < 0.001.
